# Expression and localization of FRMD7 in human fetal brain, and a role for F-actin

**Published:** 2011-02-24

**Authors:** Jiali Pu, Yingzhi Li, Zhirong Liu, Yaping Yan, Jun Tian, Sheng Chen, Baorong Zhang

**Affiliations:** 1 Department of Neurology (Key Laboratory of Cancer Prevention and Intervention, China National Ministry of Education), 2nd Affiliated Hospital, School of Medicine, Zhejiang University, Hangzhou, Zhejiang, China; 2 Department of Neurosurgery, 2nd Affiliated Hospital, School of Medicine, Zhejiang University, Hangzhou, Zhejiang, China

## Abstract

**Purpose:**

FERM domain containing 7 (FRMD7) is a member of the four-point-one, ezrin, radixin, moesin (FERM) family of proteins, and has been reported to cause X-linked idiopathic congenital nystagmus (ICN), a disease which affects ocular motor control. There have been over 30 mutations reported for FRMD7, however, their role in the pathogenesis of ICN remains unclear. The purpose of this study is to perform the expression distributes of protein FRMD7 from human fetal brain during development and to understand the relationship with cytoskeletal protein F-actin between wild-type and mutation-type FRMD7.

**Methods:**

Expression of protein FRMD7 from developing human fetal brain was tested by immunohistochemistry. Enhanced green fluorescent protein (EGFP)-tagged recombinant plasmids DNA encoding the normal or mutant FRMD7 were used to transiently transfect the mouse neuroblastoma cells (Neuro-2a) and human embryonic kidney 293 cells (HEK293T). Further, confocal microscopic analysis was used to determine the subcellular localization of the fusion proteins. To visualize F-actin, fixed HEK293T cells were stained with rhodamine-phalloidin.

**Results:**

We show that expression of FRMD7 was mainly detected in the brainstem (a region associated with ocular motor control), while limited level was observed in the cortex. The COOH-terminus of FRMD7 was found to play a key role in the subcellular localization of FRMD7 in mouse neuroblastoma cells (Neuro-2a) and human embryonic kidney 293 cells (HEK293T). While no differences in the co-localization of F-actin between the wild-type and missense mutation-type (c.781C>G and c.886G>C) proteins was observed, an additional mutant, c.1003C>T, which results in a COOH-terminally truncated protein, exhibited a nuclear localization pattern which did not co-localize with the cytoplasmic distribution of F-actin.

**Conclusions:**

The results of the present study indicate that FRMD7 may play an important role in the brainstem in the early stages of development of the human fetal brain, and provides clues for the mechanism of mutation FRMD7, which may be involved in influencing F-actin dynamics.

## Introduction

Idiopathic congenital nystagmus (ICN) is an oculomotor disorder that results from defects in the regions of the brain responsible for ocular motor control. This disorder is characterized by involuntary bilateral ocular pendular oscillations that occur within six months of birth, and can be sustained over a lifetime. As a result, patients with ICN experience a significant decrease in their quality of life [1]. Currently, the incidence of ICN is estimated to be 24 cases/10,000, and there is no effective treatment available [2].

Mutations in the *FRMD7* gene are a main cause of ICN, and more than 30 different mutations have been reported [3-9]. The *FRMD7* gene consists of 12 exons, encodes 714 amino acids, and shares a four-point-one, ezrin, radixin, moesin (FERM) domain at its NH_2_-terminus with band 4.1, ezrin, moesin, radixin, talin, filopodin, and merlin. As a result, FRMD7 has been shown to regulate the adhesion and morphogenesis of cells by modulating changes in the cytoskeleton [10,11].

Eye movement is controlled by some brain regions, such as the cortex, brainstem, and cerebellum [12,13]. In situ hybridization studies have shown that *FRMD7* mRNA is mainly expressed in the cortex plate at the early fetal cortex in humans, and at the brain cortex in mice [14,15]. For example, an evaluation of five developmental stages of the human embryonic brain (including Carnegie stages 15, 16, 19, 22, and 23), as well as two developmental stages of the human fetal brain (9- and 14-weeks post-conception [wpc]), identified strong hybridization signals associated with the cortex [14]. There are other regions of the brain that also affect eyeball movement, however, expression of FRMD7 has not been characterized in those areas.

When FRMD7 was knocked down in Neuro-2a cells, altered development of neurites was observed during retinoic acid-induced differentiation [14]. A notable increase in the dynamics of F-actin and F-actin/G-actin were also observed when FRMD7 was down-regulated in Neuro-2a cells [14]. Therefore, it has been postulated that FRMD7 regulates neuronal development by influencing the dynamics of F-actin. However, the precise function of FRMD7, and in particular its involvement in ICN pathogenesis, remain incompletely characterized. Therefore, in the present study, the expression and localization of FRMD7 was investigated in the developing human fetal brain. These studies included both wild-type FRMD7, and three mutated versions of FRMD7, which elucidated a role for F-actin in the effects of FRMD7.

## Methods

### Study protocol

The study protocol was approved by the Department of Medicine of the Second Affiliated Hospital, Zhejiang University (Hangzhou, Zhejiang Province, China). Written informed consent was also obtained from all mothers participating in the study. All samples were obtained and used in a manner compliant with the Code of Ethics of the World Medical Association (Declaration of Helsinki).

### Preparation of human tissue

Human fetal brains at stages 16–17, 21, and 25 wpc were collected from the Department of Medicine, Second Affiliated Hospital, Zhejiang University. Fresh human fetal brain tissues were fixed in 10% formalin (pH 7.2) for 24 h at room temperature (RT) before analysis.

### Immunohistochemistry

Expression and localization of FRMD7 was detected using a MaxVision^TM^ Horseradish Peroxidase-Polymer Anti-Rabbit IHC Kit (KIT-5004; Maixin-Bio, Fujian, China) according to the manufacturer’s protocol. After fixing and embedding brain samples in paraffin, 4 µm sections were cut, placed on poly-L-lysine-coated slides, and dried for 1 h at 60 °C. Brain sections were then deparaffinized using three changes of xylene separately for 10, 5, and 3 min intervals, followed by hydration in 100%, 95%, and 75% ethyl alcohol (two changes of 3 min each). Antigen retrieval was performed by microwaving sections at 98 °C in EDTA buffer (pH 7.4) for 15 min, then cooling the samples to room temperature (RT). Tissues were incubated in 3% hydrogen peroxide solution for 10 min at RT, then blocked with goat serum for 10 min. Sections were placed in a damp box for incubation with primary anti-FRMD7 antibodies (1:200 dilution; polyclonal rabbit anti-human antibody; HPA000886; Sigma–Aldrich, St Louis, MO) for 1 h at RT. Sections were incubated with secondary antibody (goat anti-rabbit; Maixin-Bio) at for 30 min at RT. After washing with 1× TBS, sections were incubated with DAB for 5 min. Negative controls were stained with rabbit normal serum instead of primary antibodies. An Olympus microscope was used for imaging (Olympus BX60; Olympus, Tokyo, Japan).

### Reverse transcription-polymerase chain reaction (RT–PCR)

Total RNA (5 µg) was reverse transcribed using oligo dT using a reverse transcriptase from the NTera-2cell line. For PCR ampliﬁcation, speciﬁc oligonucleotide primer pairs (10 pmol each) were incubated with 2 µl cDNA template in 25 µl PCR reaction mixtures containing 2.5 µl 10× PCR buffer, 1.5 mM MgSO_4_, mixed deoxynucleotides (1 mM each), and 0.5 U KOD PLUS (TOYOBO, Osaka, Japan) polymerase. The sequences of the primers used are provided Table 1. For amplification of full-length FRMD7, PCR reactions included: 35 cycles at 94 °C for 2 min; 98 °C for 15 s; 58 °C for 40 s; 68 °C for 2 min 20 s; and a final extension step at 68 °C for 7 min. For the different domains, dilutions of cDNAs were ampliﬁed for 35 cycles at 94 °C for 2 min, 98 °C for 15 s, 60 °C for 40 s, and 68 °C for 1 min 20 s. Ampliﬁed PCR products were analyzed by 1% agarose gel electrophoresis, and β-actin (*ACTB*) mRNA served as the internal standard. FRMD7 mutations included c.781C>G, c.886G>C and c.1003C>T [8], which were produced using overlap PCR (Table 1).

**Table 1:** Primers for amplification of the different domains and mutation-type *FRMD7*.

**Name**	**Sense primers (5′-3′)**	**Antisense primers (5′-3′)**	**Product length (bp)**
Full-FRMD7	gaagatctatgctacatttaaaagtgc	ggggtaccaaagctaaaaagtaattacatggttttag	2145
FERM	gaagatctgccaccatgctacatttaaaagtgcag	gggtaccaacctgaagaaagcatggtattccac	837
FERM+FA	gaagatctgccaccatgctacatttaaaagtgcag	gggtaccaactgtcgttcatggtactgagatg	1008
△FERM	ggagatctatgctttcggaagagcccaaat	ggggtaccaaagctaaaaagtaattacatggttttag	1308
△FERM+FA	ggagatctatgtgcaggtcctcaccag	ggggtaccaaagctaaaaagtaattacatggttttag	1137
781 Upstream	gaagatctatgctacatttaaaagtgc	catctccgctggccat	795
781 Downstream	ggccagcggagatgc	ggggtaccaaagctaaaaagtaattacatggttttag	1366
886 Upstream	gaagatctatgctacatttaaaagtgc	ctggaacgcttgctgc	902
886 Downstream	gcagcaagcgttccagt	ggggtaccaaagctaaaaagtaattacatggttttag	1258
C1003T	gaagatctatgctacatttaaaagtgc	gaggtaccattcatggtactgagat	1003

### Construction of plasmids

Each PCR product was conﬁrmed by subcloning the ampliﬁed cDNAs into the pGEM-T Easy Vector System (Promega, Madison, WI) for sequencing. Full-length *FRMD7* cDNA, as well as a 1,308 bp fragment encoding the △FERM domain of FRMD7 (amino acids [aa] 280–714) and a 1,137 bp fragment encoding the △FERM+FA (FERM Adjacent domain) domain of FRMD7 (aa 337–714), were digested with BglII and KpnI, and fused to enhanced green fluorescent protein (EGFP) in pEGFP-N1 (Invitrogen, Carlsbad, CA). Mutated versions of FRMD7 (c.781C>G, c.886G>C and c.1003C>T) were also subcloned into pEGFP-N1. Alternatively, the 837 bp fragment encoding the FERM domain of FRMD7 (aa 1–279) and the 1,008 bp fragment encoding the FERM and FA domains of FRMD7 (aa 1–336) were subcloned into the BglII and KpnI sites of pEGFP-C1 (Invitrogen) and fused to an NH_2_-terminal EGFP.

### Cell culture and transient transfection

Mouse neuroblastoma cells (Neuro-2A) and human embryonic kidney 293 cells (HEK293T) were purchased from the Chinese Academy of Sciences Committee Type Culture Collection Cell Bank/CAS Shanghai Institutes for Biologic Sciences Cell Resource Center (Shanghai, China). HEK293T cells and Neuro-2A cells were cultured in Dulbecco’s modified Eagle’s medium (DMEM; Invitrogen, Carlsbad, CA) containing 10% fetal bovine serum (FBS; Invitrogen), 10% penicillin, and streptomycin. Cultures were maintained in 5% CO_2_ at 37 °C, and were passaged every 2–3 days. For immunocytochemical analyses, cells were grown on chamber slides to 60%–70% conﬂuence in 24-well plates then transfected with 0.4 µg plasmid DNA and 1.5 µl Attractene Transfection Reagent (Qiagen, Valencia, CA) per well. After 6 h, cells were rinsed and fresh DMEM (containing 10% FBS) was added.

### Immunofluorescence and F-actin staining

Twenty-four hours after transfection, cells were ﬁxed in 4% formaldehyde in PBS (50 mM NaPi, 150 mM NaCl, pH 7.5) for 15 min at RT. After three washes in PBS, cell nuclei were stained with 4',6-diamidino-2-phenylindole (1:5,000 dilution; DAPI; ZhongShan Goldenbridge Biotechnology CO.,LTD, Beijing, China) for 5 min at RT. F-actin was stained using TRITC-conjugated rhodamine–phalloidin (77481; Sigma-Aldrich, St. Louis, MO) fluorescein diluted in PBS and BSA for 45 min at 37 °C. Morphological features were quantiﬁed using a confocal laser scanning microscope (Leica TCS SP5 X; Leica, Wetzlar, Germany).

## Results

### Expression of FRMD7 in the human fetal brain

**Figure 1:**
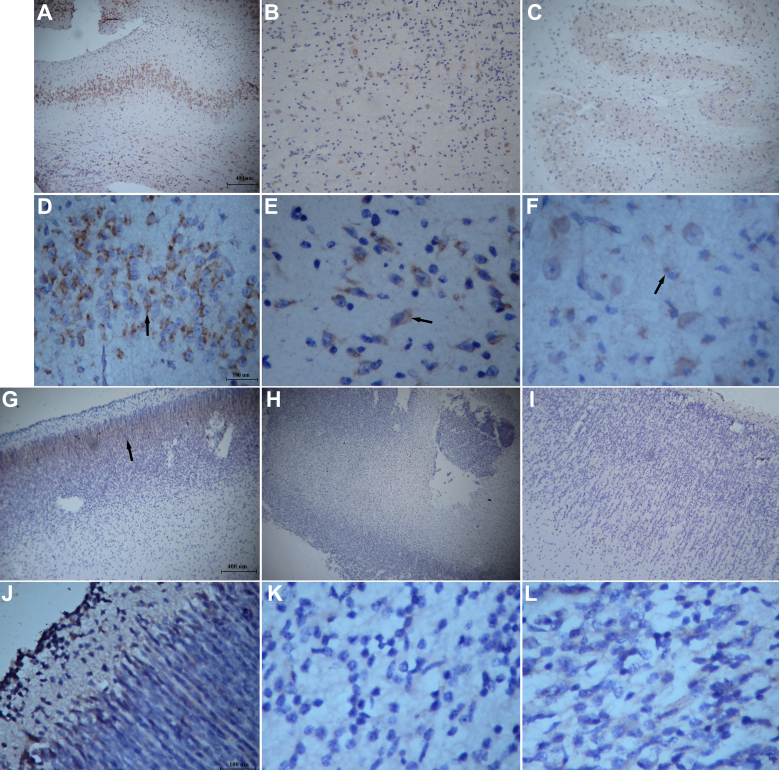
Expression of FRMD7 protein. **A**-**F**: FRMD7 protein expression in the brainstem of developing human fetal brain. Sections from human fetal brainstem tissue were examined by immunohistochemistry with anti-FRMD7 antibody. Positive staining (brown) was showed in the cytoplasm (arrows), and strong immunoreactivity was detected at 16–17 weeks post-conception (wpc) (**A**: 100×; **D**: 400×), while lower levels of FRMD7 immunoreactivity were observed at 21 wpc (**B**: 100×, **E**: 400×) and 25 wpc (**C**: 100×; **F**: 400×). Scale bars: (**A**, **B**, and **C**; 400 μm), (**D**, **E**, and **F**; 100 μm). **G**-**L**: Immunohistochemical staining of protein FRMD7 in the cortex of human fetal brain tissue using anti-FRMD7 antibody. Limited expression (arrow) was manifested in the cortex plate at 16–17 weeks post-conception (wpc; **G**: 100×; **J**: 400×), and at 21 wpc (**H**: 100×, **K**: 400×) and 25 wpc (**I**: 100×; **L**: 400×) there were little positive staining detected. Scale bars: (**G**, **H**, and **I**; 400 μm), (**J**, **K**, and **L**; 100 μm).

Expression of the nystagmus-related FRMD7 protein was examined during three stages of human fetal development (16–17, 21, and 25 wpc) and in five different regions (cortex, brainstem, hippocampus, cerebellum, and diencephalons) for each stage. At 16–17 wpc, FRMD7 was observed to be highly expressed in the pons, medulla oblongata, and midbrain (brainstem; Figure 1A-F), which are important regions associated with ocular motor control. FRMD7 was also observed in the cerebellar region and diencephalons at 16–17 wpc. During the later stages of fetal development (i.e., 21 and 25 wpc), overall expression of FRMD7 was found to significantly decrease in the brainstem, and from the cortex, limited expression was manifested in the cortex plate (Figure 1G-L).

### Subcellular localization of various forms of FRMD7 in HEK293T and Neuro-2a cells

**Figure 2:**
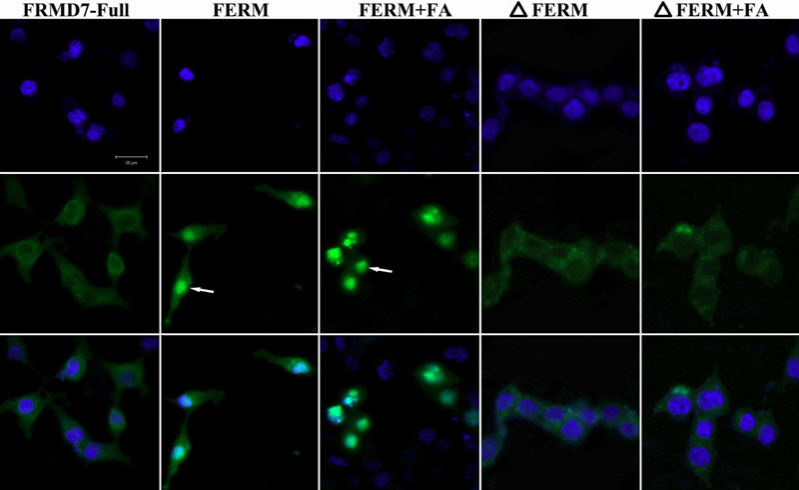
Subcellular localization of full-length FRMD7 versus various domains of FRMD7. HEK293T cells transiently transfected with EGFP-fusion proteins of FRMD7 (green) were stained with DAPI (blue) and imaged. Full-length FRMD7 was diffusely localized to the cytoplasm, while the NH_2_-terminal FERM domain (aa 1–279), and the FERM domain plus the FA domain, primarily localized to the nucleus (arrows). Constructs containing the COOH-terminal region of FRMD7 (e.g., △FERM and △FERM+FA) exhibited an expression pattern similar to that of full-length FRMD7, however, the expression level was decreased and some aggregates of these truncated proteins were detected in the cytoplasm. The results obtained from Neuro-2a cells (not shown) was the same as HEK293T cells. FERM: four-point-one, ezrin, radixin, moesin; FERM+FA: FERM domain and FERM Adjacent domain; △FERM: truncated NH_2_-terminal FERM domain; △FERM+FA; truncated NH_2_-terminal FERM domain and FERM Adjacent domain. Scale bars: 20 μm.

To better understand the function of FRMD7, HEK293T and Neuro-2a cells were transiently transfected with various EGFP-tagged fragments of FRMD7. In these experiments, full-length FRMD7 was found to be uniformly distributed in the cytoplasm. In contrast, when various regions of FRMD7 were overexpressed, only the COOH-terminal regions of FRMD7 (△FERM and △FERM+FA) resulted in a distribution pattern that was similar to that of full-length FRMD7. However, for these modified FRMD7 proteins, the expression levels were lower, and some aggregates were observed in the cytoplasm. For FRMD7 constructs containing only the NH_2_-terminal FERM domain (aa 1–279) of FRMD7, or a combination of the FERM domain and the FA domain (adjacent to the FERM domain; aa 294–336), a predominantly nuclear distribution was observed (Figure 2).

### Role of F-actin dynamics

**Figure 3:**
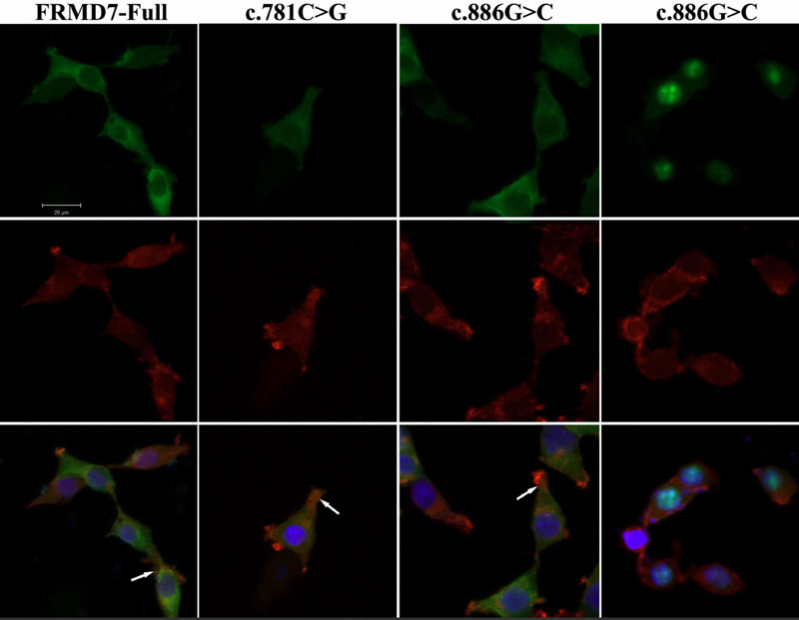
Co-localization of F-actin and FRMD7. HEK293T cells transiently transfected with EGFP-tagged wild-type FRMD7 and FRMD7 mutants, c.781C>G and c.886G>C, (shown in green) were stained with TRITC-conjugated rhodamine–phalloidin fluorescein (red). All three fusion proteins exhibited a diffuse localization pattern in the cytoplasm, and also in actin-rich regions of the HEK293T cells (arrows). However, HEK293T cells expressing the FRMD7 mutant, c.1003C>T, fused to EGFP, primarily exhibited localization of the fusion protein to the nucleus and did not co-localize with F-actin (merge). Scale bars: 20 μm.

It is hypothesized that FRMD7 regulates neuronal development by influencing F-actin dynamics. Therefore, EGFP-tagged mutated versions of FRMD7 were transiently transfected into HEK293T cells and these cells were stained with TRITC rhodamine–phalloidin to detect F-actin. In these experiments, both wild-type FRMD7 and c.781C>G and c.886G>C FRMD7 mutants exhibited a diffuse distribution in the cytoplasm, and also in actin-rich regions. However, another FRMD7 mutant, c.1003C>T, exhibited changes in its subcellular distribution pattern and did not co-localize with F-actin (Figure 3).

## Discussion

FRMD7 is related to the X-linked ICN. *FRMD7* mRNA has recently been shown to have a temporal and spatial expression pattern in the cerebral cortex in the developing human embryonic brain and early fetal brain. However, the expression pattern in other brain regions also related to eyeball movement and in later development stages of human fetal brain were not known. In the present study, expression of FRMD7 was detected during three different stages of human fetal development and in five specific regions of the brain. From these experiments, it was observed that expression of FRMD7 is not restricted to the cortex, since at 16–17 wpc, FRMD7 is highly expressed in the pons, medulla and oblongata. Moreover, moderate expression of FRMD7 was detected in the cerebellar region and diencephalons. The highest levels of expression that were detected correlate with regions of the brain associated with ocular motor control. In the cortex, limited expression of FRMD7 in the cortex plate was also observed, which suggests a role for FRMD7 in the brainstem. Furthermore, in the later stages of fetal development (e.g., 21 and 25 wpc), overall expression of FRMD7 was significantly lower in the brainstem and cortex. Therefore, since expression of FRMD7 was not detected in the cerebellar region and diencephalons in the later stages of development, we hypothesize that FRMD7 mainly affects the brainstem during early development of the fetal brain.

FRMD7 is a member of the FERM family of proteins, and for a subset of these proteins, subcellular distribution has been shown to be critical for their function (e.g., merlin, moesin, and FAK) [16,17]. Studies have also shown that the subcellular localization of most FERM family proteins is not dependent on the NH_2_-terminal FERM domain, but rather on the COOH-terminal domains of FRMD7 [17]. Correspondingly, in this study, only constructs containing the COOH-terminal FERM domain (e.g., △FERM and △FERM+FA) produced an expression pattern similar to that of full-length FRMD7, while proteins lacking the COOH-terminal domain, yet maintaining the NH_2_-terminal FERM domain, or containing the FERM and FA domains, exhibited a primarily nuclear distribution. In combination, these data suggest that the COOH-terminus of FRMD7 plays a significant role in determining the subcellular localization of FRMD7.

When FRMD7 was knocked down in Neuro-2a cells, a signiﬁcant reduction in the overall length of neurites was detected, along with an increase in levels of F-actin and changes in the dynamics between F-actin and G-actin [14]. Based on these results, a unique role for FRMD7 in association with F-actin was identified. However, the role of FRMD7 mutations in ICN is incompletely understood. Of the mutation sites identified, most are localized in the NH_2_-terminal FERM domain of FRMD7. However, it is unclear how mutations present in different domains eventually lead to the same disease. We previously reported two missense mutations, c.781C>G and c.886G>C, which lead to an arginine in the protein 261 loci being substituted for glycine (p.R261G), and glycine in the protein 296 loci being substituted for arginine (p.G296R), respectively. A nonsense mutation (c.1003C>T) was also identified, which results in the arginine of the protein 335 loci to be substituted for a stop codon (p.R335X) [8]. Therefore, in this study, rhodamine–phalloidin staining was used to further characterize the role of F-actin in relation to these three mutations. In HEK293T cells, both wild-type and c.781C>G and c.886G>C mutants were observed to co-localize with F-actin. However, the c.1003C>T mutant of FRMD7 (which produces a COOH-terminal truncated protein), did not co-localize with F-actin. These findings suggest that FRMD7 may mediate regulation of the cytoskeleton. Although the c.781C>G and c.886G>C mutations did not alter the co-localization of FRMD7 and F-actin, the FERM domain has previously been associated with regulation of the cytoskeleton. Moreover, the role for FRMD7 in neuronal development is similar to that of other FERM family proteins, particularly FARP1 and FARP2 [11,18,19], where their regulation of the cytoskeleton is mediated by the activation of small GTPases. Therefore, the role of FRMD7 in neuronal development may involve the cytoskeleton.

In summary, the present study demonstrates that FRMD7 is mainly expressed in the brainstem during the early stages of development of the human fetal brain. The subcellular localization of FRMD7 is also shown to be dependent on COOH-terminal regions of FRMD7, as well as the type of mutation(s) present (e.g., location of the mutant or protein truncation). For the c.1003C>T mutant of FRMD7, truncation of the COOH-terminus of FRMD7 alters the relationship between F-actin and FRMD7. Overall, these ﬁndings indicate that additional studies are needed to investigate the function and mechanism of FRMD7 in neuronal development, particularly as it relates to the pathogenesis of ICN.
